# Hypercapnic acidosis affects *in vitro* lung infection response in a pathogen-specific manner

**DOI:** 10.3389/fcimb.2025.1708427

**Published:** 2026-02-03

**Authors:** Elena Campaña-Duel, Aina Areny-Balagueró, Luis Morales-Quinteros, Laia Fernández-Barat, Diana Fuertes-Bailón, Antoni Torres, Lluís Blanch, Antonio Artigas, Adrian Ceccato, Marta Camprubí-Rimblas

**Affiliations:** 1 Critical Care Research Center, Parc Taulí Hospital Universitari, Institut d’Investigació i Innovació Parc Taulí (I3PT-CERCA) and Universitat Autònoma de Barcelona, Sabadell, Spain; 2 Centro de Investigación Biomédica En Red de Enfermedades Respiratorias, Instituto de Salud Carlos III, Barcelona, Spain; 3 Servei de Medicina Intensiva, Hospital Universitari Vall d’Hebron - Vall d’Hebron Institut de Recerca, Barcelona, Spain; 4 CELLEX research laboratories, Institut d'Investigacions Biomèdiques August Pi i Sunyer (IDIBAPS), Barcelona, Spain; 5 Faculty of Medicine & Faculty of Pharmacy and food Sciences, University of Barcelona, Barcelona, Spain; 6 Pulmonology Department, Hospital Clínic, Barcelona, Spain; 7 Research Support Unit, Parc Taulí Hospital Universitari, Institut d’Investigació i Innovació Parc Taulí (I3PT-CERCA) and Universitat Autònoma de Barcelona, Sabadell, Spain; 8 Servei de Medicina Intensiva, Corporació Sanitària i Universitària Parc Taulí, Sabadell, Spain; 9 Hospital Universitari Sagrat Cor Grupo Quironsalud, Barcelona, Spain

**Keywords:** alveolar epithelial cell, CO2, hypercapnia, lung infection, macrophage

## Abstract

**Background:**

Hypercapnic acidosis (HCA) is a hallmark of acute hypercapnic respiratory failure, often triggered by respiratory infections. Its role in lung injury remains controversial, with both protective and detrimental effects reported. However, the specific impact on pulmonary immune responses to lung infections remain poorly understood. To investigate the impact of HCA on alveolar response during infection, we developed an *in vitro* model combining human alveolar epithelial cells (type I and II) and macrophage-like THP-1 cells.

**Methods:**

The co-culture and monocultures were infected with *Pseudomonas aeruginosa* or *Streptococcus pneumoniae* under normocapnic or HCA conditions. At 1 hour and 24 hours post-infection, we assessed inflammatory cytokine expression (IL-1β, CCL2, IL-8), tight junction protein levels (occludin, ZO-1) and bacterial survival.

**Results:**

HCA modulated inflammation in pathogen-specific manner: IL-1β induction by *S. pneumoniae* was mainly CO_2_-driven, while *P. aeruginosa* triggered strong IL-1β regardless of CO_2_. Tight junction proteins were upregulated at 1 hour under HCA, particularly with macrophages, but occludin declined at 24 hours, potentially impairing epithelial repair. While extracellular bacterial loads were unaffected by CO_2_, HCA promoted intracellular replication of *P. aeruginosa* in macrophages, without affecting intracellular survival in epithelial cells or overall bacterial burden in *S. pneumoniae*-infected cultures.

**Conclusions:**

HCA condition differentially influence host responses depending on the infectious pathogen, compromising the barrier function and prolonging lung inflammation with differences according to time culture, which could benefit bacterial persistence.

## 1 Background

Acute or chronic lung diseases might result in hypercapnia, defined as an increase in the partial pressure of carbon dioxide (PaCO_2_) above 45 mmHg (Kavanagh and Laffey, 2006; Almanza-Hurtado et al., 2022). When accompanied by a blood pH below 7.35, it is termed as hypercapnic acidosis (HCA), a hallmark of acute hypercapnic respiratory failure (Mirabile et al., 2025). Episodes of acute hypercapnia are often triggered by respiratory infections and associated lung damage (Papi et al., 2006; Celli and Barnes, 2007).

The impact of hypercapnia in respiratory diseases, with or without acidosis, has been studied in models of ventilator-induced lung injury (VILI), acute lung injury, and infection (Shibata et al., 1998; Laffey et al., 2000; Sinclair et al., 2002; Vadász et al., 2012; Campaña-Duel et al., 2024), where its role as a protective or detrimental factor remain controversial. Evidence suggests that HCA has beneficial effects by reducing inflammation, inhibiting NF-κβ signalling, and reducing oxidative damage in the alveoli (Contreras et al., 2012). However, the same pathway inhibition impairs bacterial clearance and reduces macrophage phagocytic activity (Wang et al., 2010). Its effects on alveolar epithelial cells during infection are less understood, although studies on *in vitro* and *ex vivo* VILI models suggest that hypercapnia hampers wound healing and lung repair (Doerr et al., 2005; Briva et al., 2007; Cortes-Puentes et al., 2019).

Despite these findings, the effects of HCA on bacterial lung infections remain poorly understood. As lower respiratory tract infections (LRTI) are a leading cause of global morbidity and mortality (GBD 2021 Lower Respiratory Infections and Antimicrobial Resistance Collaborators, 2024), it is crucial to understand how HCA modulates alveolar responses to infection, including cell death, barrier dysfunction, immune cell infiltration, oedema, and impaired gas exchange.

To address this, we developed an *in vitro* co-culture of alveolar epithelial cells (type I and II) and macrophages, mimicking the first line of alveolar defence. This model better reflects the alveolar microenvironment than monocultures and allows investigation of HCA effects during infection by two clinically relevant pathogens: *Pseudomonas aeruginosa* and *Streptococcus pneumoniae*.

This study hypothesizes that HCA modulates the inflammation, tight junction integrity, and bacterial survival in the proposed co-culture model during bacterial infection. To test this, we analysed inflammatory profile, tight junction expression and bacterial survival under HCA versus normocapnic conditions. These outcomes were evaluated at different time points post-infection in both co-culture and monocultures to dissect the individual and combined contributions of the involved cellular types.

## 2 Methods

### 2.1 Cell cultures

**Figure 1:**
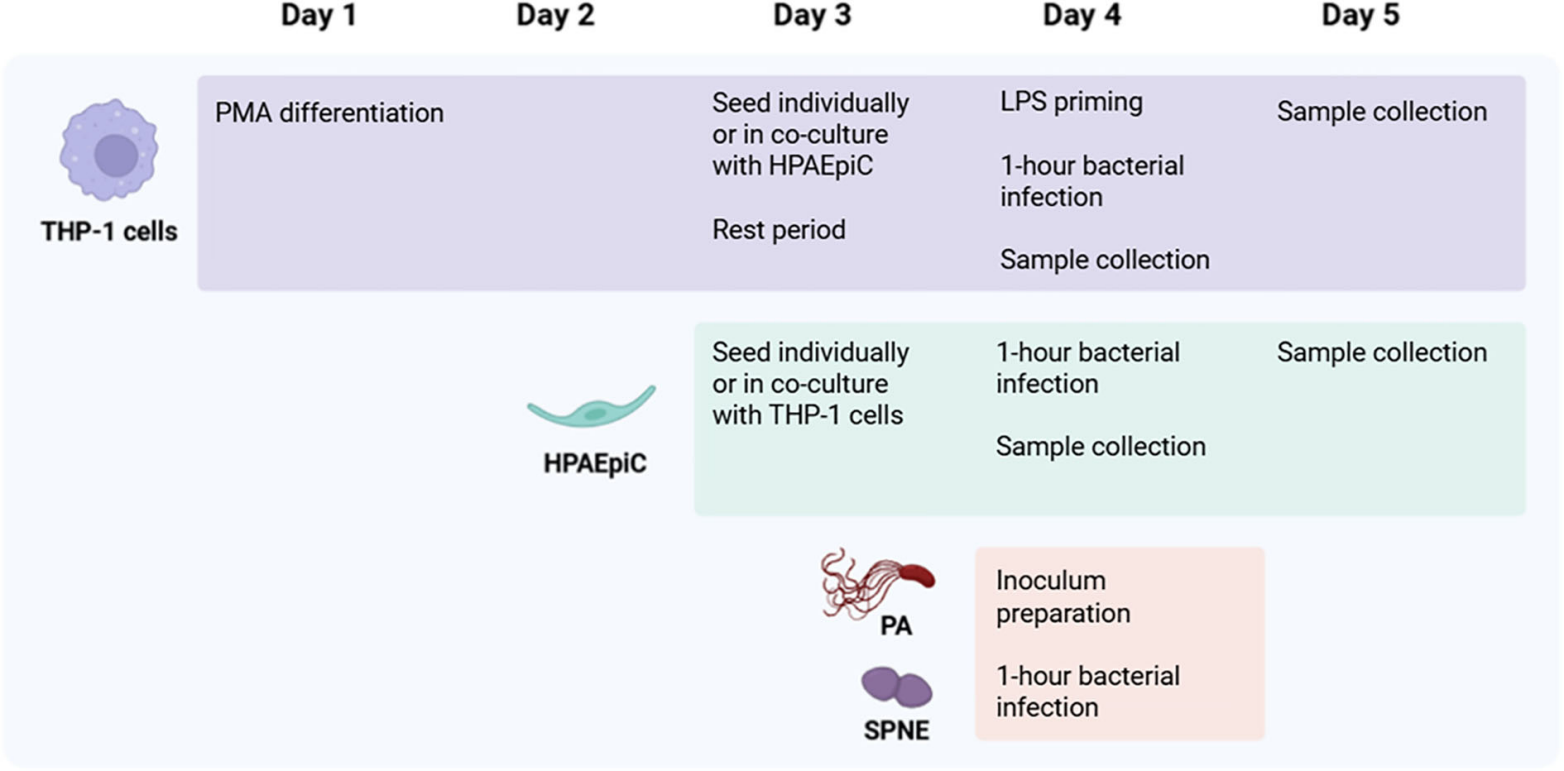
Schematic experimental design. Diagram illustrating the experimental setup, including the cell types, bacterial infection, and time points analyzed. Created with BioRender.com. PA: *P. aeruginosa*; SPNE: *S. pneumoniae*; HPAEpiC: Human Pulmonary Alveolar Epithelial Cells; PMA: Phorbol 12-Myristate.

Human monocytic THP-1 cells (American Type Culture Collection, Virginia, USA) and human pulmonary alveolar epithelial cells (HPAEpiC) (Innoprot, Bizkaia, Spain) were seeded in 24-well plates, either in monoculture or co-culture at a 1:5 ratio (**Figure 1**) (Fritz et al., 2011).

THP-1 cells were cultured and differentiated into macrophage-like cells with 100 ng/mL phorbol 12-myristate 13-acetate (PMA) (Sigma-Aldrich, St. Louis, MO, USA) for 48 hours in RPMI 1640 medium (Biowest, Nuaillé, France) supplemented with 10% heat-inactivated fetal bovine serum (FBS) (Innoprot, Bizkaia, Spain), 1% penicillin-streptomycin (p/s) (Capricorn Scientific, Ebsdorfergrund, Germany), 1% amphotericin (Biowest, Nuaillé, France) and 0.5% L-glutamine (Capricorn Scientific, Ebsdorfergrund, Germany). Cells were incubated 24 hours in Alveolar Epithelial Cell Medium (AEpiCM) supplemented with 2% FBS, 1% Epithelial Cell Growth Supplement (EpiCGS) (Innoprot, Bizkaia, Spain), and 1% p/s. Five hours before infection, cells were primed with AEpiCM containing 2 µg/mL lipopolysaccharide (LPS) from Escherichia coli O55:B5 (Sigma-Aldrich, St. Louis, MO, USA).

HPAEpiC (passages 4 to 6) were cultured in AEpiCM with 2% inactivated FBS, 1% EpiCGS and 1% p/s on poly-L-lysine-coated plates (Sigma-Aldrich, St. Louis, MO, USA), seeded individually or in co-culture with macrophage-like THP-1 cells seeded 48 hours earlier. Cultures were rested for 24 hours.

### 2.2 Cell infection

*Pseudomonas aeruginosa* (strain PAO1) and *Streptococcus pneumoniae* (serotype 19A) (WHO bacterial priority pathogens list, 2024) were grown to log phase in Luria Broth (LB) medium (Gibco, Thermo Fisher Scientific, Waltham, MA, USA) or THY medium (Todd Hewitt broth medium with 2% yeast extract) (Sigma-Aldrich, St. Louis, MO, USA), respectively. Cultures were infected at MOI 1:50 (P. aeruginosa) or 1:20 (S. pneumoniae) for 1 h at 37°C under normocapnic (5% CO_2_, pH 7.3) or hypercapnic (15% CO_2_, pH 7.0) conditions. Both pathogens have proven lung infection capacity in experimental models previously described (Amaro et al., 2021; Motos et al., 2023). Uninfected controls were included. Post-infection, cells were washed with PBS + 5X p/s and fresh supplemented AEpiCM was added. Samples were collected at 1 and 24 hours post-infection to obtain cell supernatant and total intracellular protein.

### 2.3 Secretome and intracellular protein analysis

Supernatants were centrifuged (500 x g, 5 minutes, RT), and IL-1β (DY201-05), CCL2 (DY279-05) and IL-8 (DY208-05) were quantified by enzyme-linked immunosorbent assay (ELISA) kits (R&D Systems, Minneapolis, USA).

Intracellular proteins were extracted using lysis buffer (see Supplementary Methods) with protease inhibitor cocktail (Roche, Basel, Switzerland) and sodium orthovanadate, and analysed by ELISA for zonula occludens 1 (ZO-1) (EH15434) and occludin (EH1674) (FineTest, Boulder, CO, USA).

### 2.4 Analysis of bacterial survival

Post-infection, extracellular bacteria were assessed by plating supernatants on blood agar plates. Intracellular bacteria were quantified after antibiotic washes (p/s, gentamicin or vancomycin), lysis with cold distilled water, and colony forming units (CFU) counting at 1, 2, 22, and 24 hours.

### 2.5 Statistical analysis

Data normality was evaluated by Shapiro-Wilk test. Depending on distribution, t-tests or Mann-Whitney tests were applied. Results were expressed as the geometric mean ± standard error of the mean (SEM). Linear mixed effects (LME) models assessed the impact of CO_2_ and infection. Results were analyzed using GraphPad Prism 8 and R (v.4.3.0) with RStudio (2024.12.1 Build 563). p ≤ 0.05 was considered significant.

Detailed methods are provided in the data supplement.

## 3 Results

### 3.1 THP-1 cell-like macrophage differentiation

Differentiation of LPS-stimulated THP-1 cells into M1 macrophages was confirmed by assessment of cell adhesion to the plate and increased expression of proinflammatory markers characteristic of the M1 phenotype, including IL-1β and CD86 compared to non-LPS-stimulated THP-1 cells (data not shown). Consequently, uninfected control cultures exhibited elevated baseline cytokine levels, reflecting this pre-activated proinflammatory state.

### 3.2 Hypercapnic acidosis increases proinflammatory mediator release early after infection

**Figure 2:**
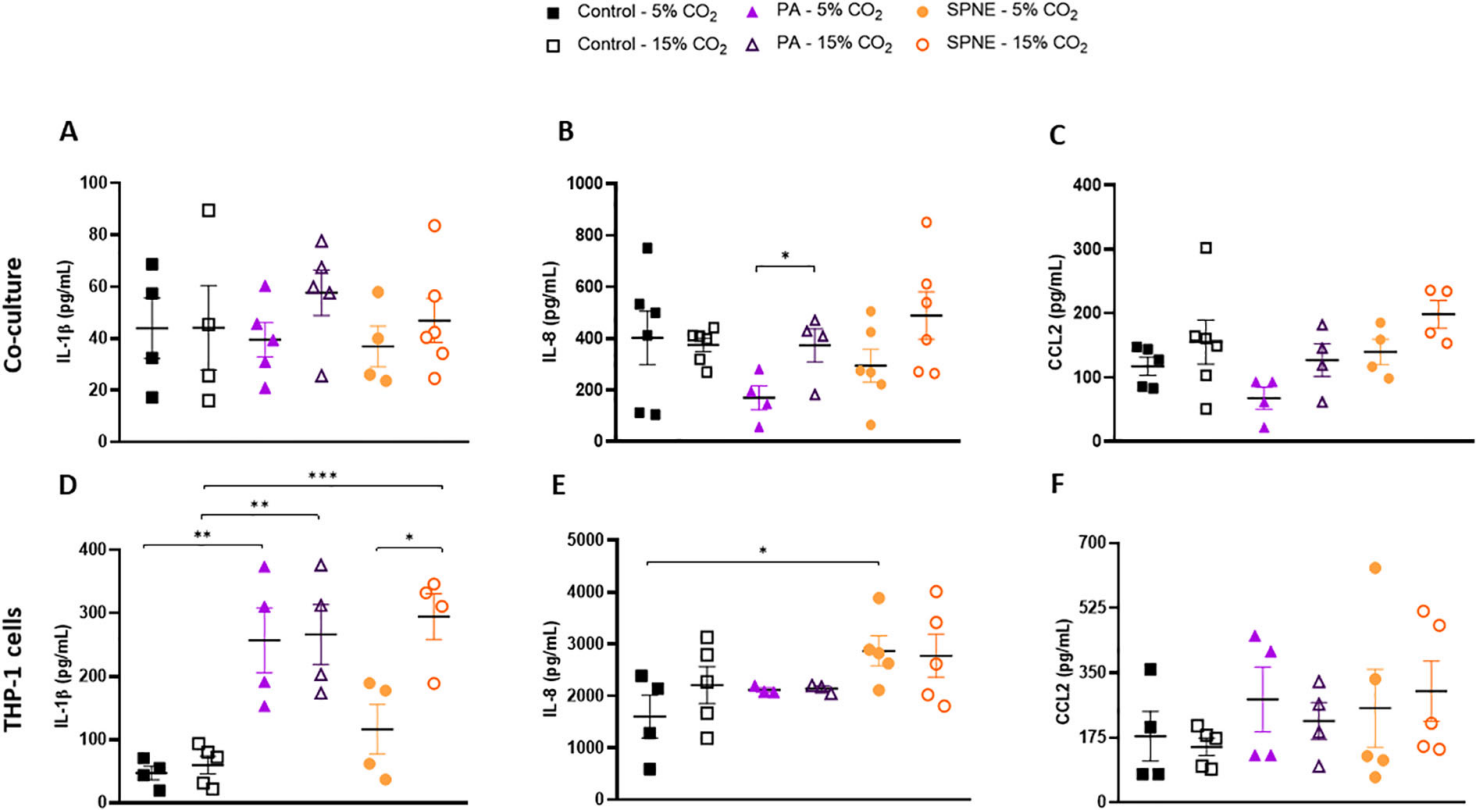
Cytokine levels in co-culture and macrophage-like THP-1 cells after 1 hour of bacterial infection under normocapnic and hypercapnic acidosis conditions. Protein concentrations of pro-inflammatory IL-1β **(A, D)**, IL-8 **(B, E)**, and chemokine CCL2 **(C, F)** in the supernatant of co-culture of HPAEpiC and macrophage-like THP-1 cells **(A-C)**, and macrophage-like THP-1 cells culture **(D-F)** after 1 hour of infection with *P. aeruginosa* (PA) or *S. pneumoniae* (SPNE) under normocapnic (5% CO_2_) and hypercapnic (15% CO_2_) conditions. n = 4-6. Data represented as mean ± SEM. * p≤0.05; ** p≤0.01; *** p≤0.001.

**Figure 3:**
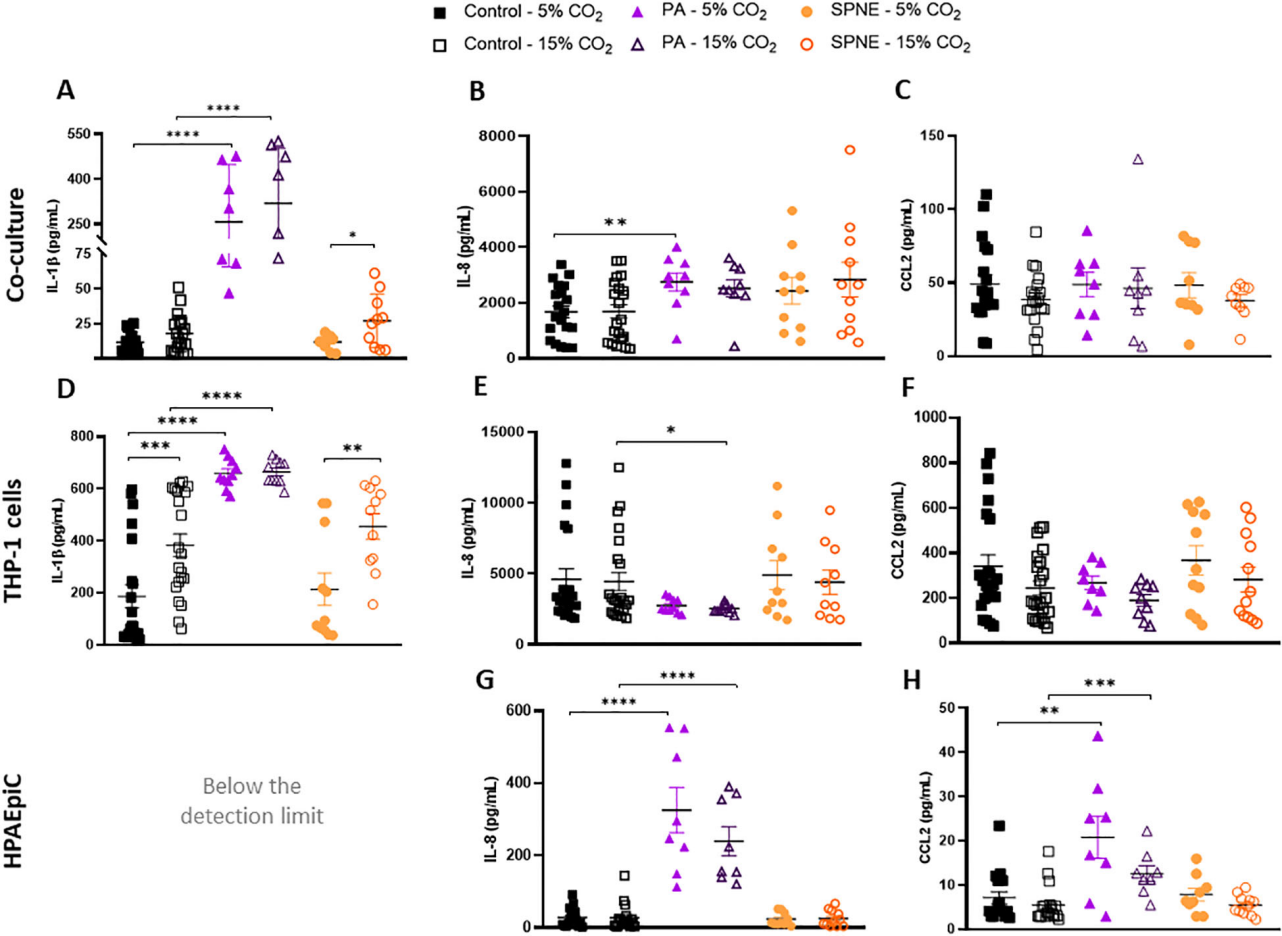
Cytokine levels in co-culture, macrophage-like THP-1 cells and HPAEpiC after 24 hours of bacterial infection under normocapnic and hypercapnic acidosis conditions. Protein concentrations of pro-inflammatory IL-1β **(A, F)**, IL-8 **(B, D, G)**, and chemokine CCL2 **(C, E, H)** in the supernatant co-culture of HPAEpiC and macrophage-like THP-1 cells **(A-C)**, macrophage-like THP-1 cells **(D-F)** and HPAEpiC **(G-H)** after 24 hours of infection with *P. aeruginosa* (PA) or *S. pneumoniae* (SPNE) under normocapnic (5% CO2) and hypercapnic (15% CO2) conditions. n = 9-12. Data represented as mean ± SEM. * p≤0.05; ** p≤0.01; *** p≤0.001; **** p≤0.0001.

One hour after the onset of infection, in the co-culture of HPAEpiC and macrophage-like THP-1 cells, no differences in IL-1β and CCL2 protein levels were detected (**Figures 2A, C**). However, IL-8 levels were significantly higher in *P. aeruginosa* infection under HCA compared to normocapnia (**Figure 2B**). No effect of HCA or interaction with the different infections was determined in the LME model (**Table 1**) CCL2 (**Figures 3B, C**; **Table 2**).

**Table 1:** Effects of CO_2_ conditions and bacterial infection on cytokine levels expressed by co-culture and macrophage-like THP-1 cells after 1 hour of infection.

Culture	Cytokine	Beta estimate for CO_2_ (95% CI)	Beta estimate for *P. aeruginosa* infection (95% CI)	Beta estimate for S. pneumoniae infection (95% CI)	Beta estimate for CO_2_ × *P. aeruginosa* interaction (95% CI)	Beta estimate for CO_2_ × *S. pneumoniae* interaction (95% CI)
Co-culture	IL-1β	-1.205 (-3.816 to 1.406)	-16.978 (-60.615 to 26.659)	-13.538 (-59.710 to 32.634)	3.019 (-0.672 to 6.710)	2.240 (-1.500 to 5.980)
Co-culture	IL-8	-3.454 (-20.357 to 13.449)	-325.434* **(-631.629 to -19.239)**	-115.582 (-408.541 to 177.377)	**25.048.** **(-20.357 to 12.449)**	**22.968.** **(-2.292 – 48.228)**
Co-culture	CCL2	0.307 (-8.836 to 9.450)	-35.086 (-187.735 to 117.563)	-39.955 (-201.582 to 121.672)	0.470 (-13.546 to 14.486)	5.555 (-8.902 to 20.012)
Macrophage-like THP-1	IL-1β	1.263 (-7.087 to 9.613)	228.98** **(91.014 to 366.958)**	-21.736 (-161.580 to 118.108)	-0.345 (-12.411 to 11.721)	16.548* **(4.482 to 28.614)**
Macrophage-like THP-1	IL-8	**91.858.** **(-4.288 to 188.004)**	**1,582.240.** **(-30.771 to 3,195.251)**	2,077.616* **(556.764 to 3,598.468)**	-94.229 (-238.446 to 49.988)	-101.273 (-237.242 to 34.696)
Macrophage-like THP-1	CCL2	-1.332 (-17.135 to 14.471)	84.022 (-177.101 to 345.145)	49.068 (-206.428 to 304.564)	-4.473 (-27.297 to 18.351)	5.942 (-15.661 to 27.545)

Beta coefficients are estimated from linear mixed-effects (LME) models including CO_2_ exposure, *P. aeruginosa* infection, *S. pneumoniae* infection, and their interaction terms as fixed effects. CO_2_ was entered as a numeric covariate, whereas *P. aeruginosa* and *S. pneumoniae* infections were included as factor variables. The models also included random effects to account for variability among biological replicates. Values are reported as beta estimates with 95% confidence intervals. n = 4–6 for each condition. Significance codes: p ≤ 0.1; *p ≤ 0.05; **p ≤ 0.01; ***p ≤ 0.001; ****p ≤ 0.0001.Statistically significant values are highlighted in bold.

In macrophage-like THP-1 cells alone, *P. aeruginosa* induced a strong proinflammatory response in terms of IL-1β protein secretion, with no differences between the CO_2_ conditions (**Figure 2D**; **Table 1**). Conversely, *S. pneumoniae* infection significantly increased IL-1β secretion only under HCA (**Figure 2D**; **Table 1**). Regarding chemokine secretion, neither normocapnic nor HCA conditions increased the levels of CCL2 and IL-8 in macrophage-like THP-1 cells infected with *P. aeruginosa*, although *S. pneumoniae* infection under normocapnia raised IL-8 levels (**Figures 2E, F**).

These biomarkers were not detected in the HPAEpiC 1 hour post-infection by ELISA technique.

### 3.3 Hypercapnic acidosis alters proinflammatory mediator in late-stage *S. pneumoniae* infection

At 24 hours post-infection, the co-cultures infected by *P. aeruginosa* significantly increased IL-1β protein level, and no effect was detected for different CO_2_ conditions (**Figure 3A**). However, in *S. pneumoniae*-infected co-cultures, HCA specifically increased IL-1β secretion (**Figure 3A**). In terms of chemokine production in the co-cultures, HCA significantly decreased the levels of CCL2 (**Table 2**).

**Table 2:** Effects of CO_2_ conditions and bacterial infection on cytokine levels expressed by co-culture, macrophage-like THP-1 cells and HPAEpiC after 24 hours of infection.

Culture	Cytokine	Beta estimate for CO_2_ (95% CI)	Beta estimate for *P. aeruginosa* infection (95% CI)	Beta estimate for S. pneumoniae infection (95% CI)	Beta estimate for CO_2_ × *P. aeruginosa* interaction (95% CI)	Beta estimate for CO_2_ × *S. pneumoniae* interaction (95% CI)
Co-culture	IL-1β	0.643 (-4.700 to 5.986)	227.247**** **(117.577 to 336.917)**	-10.167 (-116.168 to 95.834)	6.957 (-2.867 to 16.781)	0.865 (-8.606 to 10.336)
Co-culture	IL-8	-17.962 (-100.119 to 64.195)	731.297 (-977.208 to 2,439.802)	591.500 (-1,003.229 to 2,186.229)	-4.678 (-157.154 to 147.798)	-11.253 (-153.553 to 131.047)
Co-culture	CCL2	-2.003* **(-3.910 to -0.096)**	-5.632 (-45.275 to 34.011)	0.063 (-36.940 to 37.066)	1.012 (-2.526 to 4.550)	0.973 (-2.330 to 4.276)
Macrophage-like THP-1	IL-1β	15.973**** **(8.688 to 23.258)**	505.211**** **(356.600 to 653.822)**	-4.145 (-143.523 to 135.233)	-15.419* (-28.653 to -2.185)	-0.498 (-12.940 to 11.944)
Macrophage-like THP-1	IL-8	-65.114 (-207.728 to 77.500)	-2,148.882 (-5,057.491 to 759.727)	1,602.747 (-1,125.469 to 4,330.963)	43.192 (-215.879 to 302.263)	-71.877 (-315.436 to 171.682)
Macrophage-like THP-1	CCL2	-4.404 (-22.156 to 13.348)	55.350 (-306.172 to 416.872)	145.132 (-194.248 to 484.512)	-14.352 (-46.600 to 17.896)	-13.436 (-43.753 to 16.881)
HPAEpiC	IL-8	-12.385 (-33.122 to 8.352)	205.364 (-224.930 to 635.658)	**-**200.726 (-602.303 to 200.851)	4.070 (-34.417 to 42.557)	12.724 (-23.193 to 48.641)
HPAEpiC	CCL2	0.063 (-0.925 to 1.051)	33.585** **(13.046 to 54.124)**	5.782 (-13.389 to 24.953)	-1.390 (-3.225 to 0.445)	-0.488 (-2.201 to 1.225)

Beta coefficients are estimated from linear mixed-effects (LME) models including CO_2_ exposure, *P. aeruginosa* infection, *S. pneumoniae* infection, and their interaction terms as fixed effects. CO_2_ was entered as a numeric covariate, whereas *P. aeruginosa* and *S. pneumoniae* infections were included as factor variables. The models also included random effects to account for variability among biological replicates. Values are reported as beta estimates with 95% confidence intervals. n = 9–12 for each condition. Significance codes: p ≤ 0.1; *p ≤ 0.05; **p ≤ 0.01; ***p ≤ 0.001; ****p ≤ 0.0001.Statistically significant values are highlighted in bold.

The macrophage-like THP-1 cells infected with *P. aeruginosa* exhibited a robust IL-1β response compared to the non-infected controls (**Figure 3D**). HCA had a significant increase on IL-1β levels (**Table 2**), but not in the presence of *P. aeruginosa* (**Figure 3B, C**; **Table 2**). Regarding chemokine production, *P. aeruginosa*-infected macrophage-like THP-1 cells had lower IL-8 levels, and this difference was significant under HCA (**Figure 3E**), but not in the LME model. Overall, HCA did not significantly affect chemokine expression by macrophages 24 hours after infection (**Figures 3E, F**; **Table 2**).

Conversely, in *P. aeruginosa*-infected HPAEpiC cells, a significant increase in chemokine production (IL-8 and CCL2) was detected, although this response seemed to be more pronounced under normocapnic conditions. However, the difference between the two CO_2_ conditions was not statistically significant (**Figures 3G, H**). Neither HCA nor *S. pneumoniae* infection had any effect on chemokine production by HPAEpiC.

### 3.4 Differential response of tight junction components to hypercapnia

**Figure 4:**
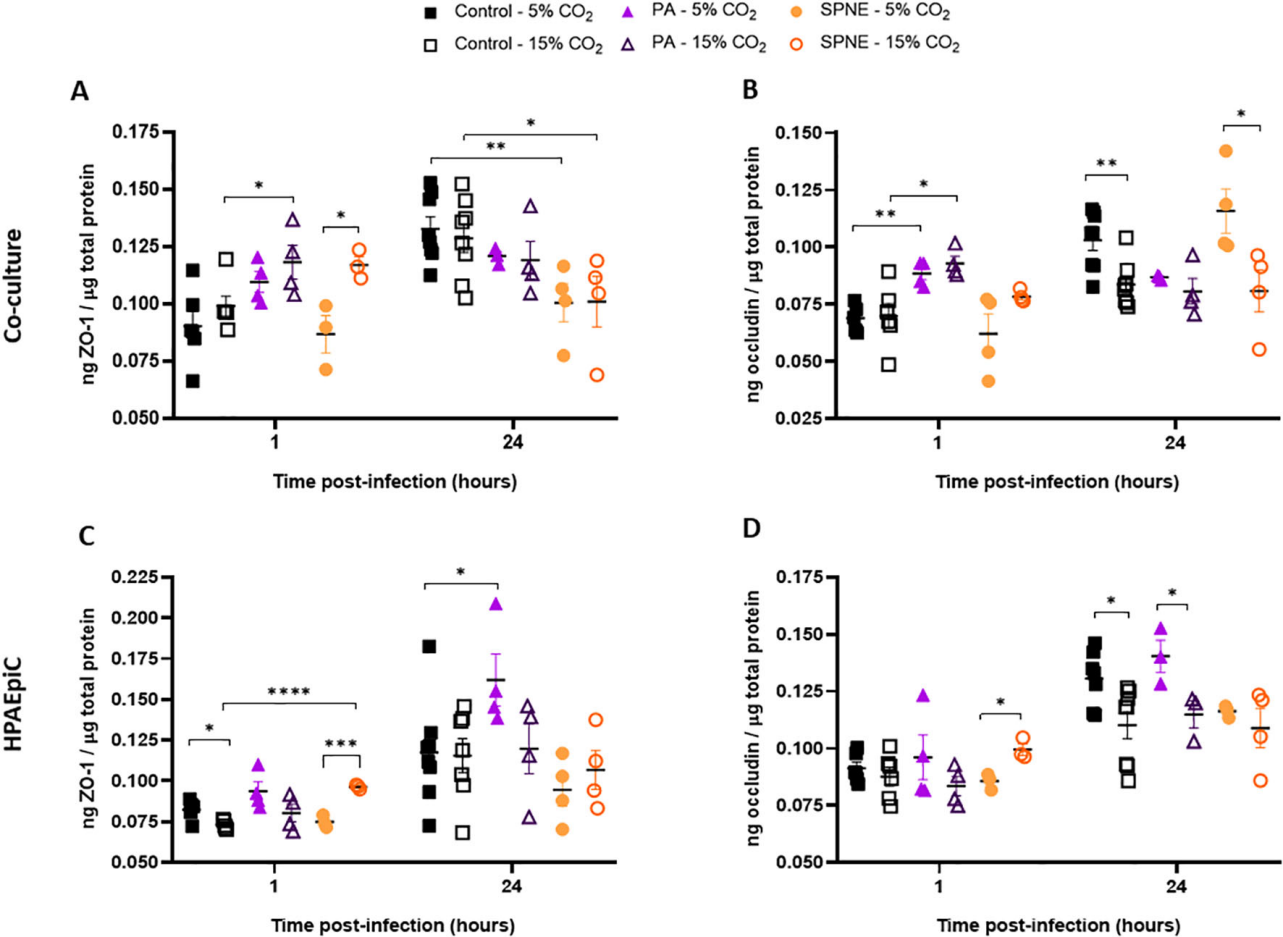
Tight junction protein measurement in co-culture cells and HPAEpiC after 1 and 24 hour of bacterial infection under normocapnic and hypercapnic acidosis conditions. Protein concentration in cell culture supernatant of zonula occludens (ZO-1) **(A, C)** and occludin **(B, D)** in total intracellular protein 1 hour and 24 hours post-infection with *P. aeruginosa* (PA) or *S. pneumoniae* (SPNE) for HPAEpiC and macrophage-like THP-1 cell co-cultures (A, B) and HPAEpiC monocultures **(C, D)**. n = 4-6. Data are presented as mean ± SEM. * p ≤ 0.05; ** p ≤ 0.01; *** p ≤ 0.001; **** p ≤ 0.0001.

In the co-culture at 1 hour post-infection, *P. aeruginosa* infection under HCA significantly increased ZO-1 and occludin expression compared to non-infected cells. In the case of *S. pneumoniae*, although no increase was observed compared to non-infected-cells, HCA led to a higher ZO-1 expression in infected cells (**Figures 4A, B**). At the 24-hour time point, both *P. aeruginosa*- and *S. pneumoniae*-infected cultures did not differ in ZO-1 levels between normocapnic and HCA conditions (**Figure 4A**). However, a marked reduction in occludin production was found, particularly in the uninfected cultures and *S. pneumoniae*-infected groups when submitted to an hypercapnic environment (**Figure 4B**).

In HPAEpiC cultures infected with *S. pneumoniae* under HCA, a significant increase in both intracellular junction proteins was observed at 1 hour post-infection compared to the normocapnic condition (**Figures 4C, D**). In contrast, at 24 hours post-infection, no significant differences in ZO-1 levels were detected between the same experimental groups exposed to 5% or 15% of CO_2_ (**Figures 4C, D**). Meanwhile, occludin levels were significantly decreased in both control and *P. aeruginosa*-infected HPAEpiC cultures under HCA when compared to normocapnia (**Figure 4D**).

### 3.5 Hypercapnic acidosis promotes higher intracellular *P. aeruginosa* burden in macrophage-like THP-1 cells

**Figure 5:**
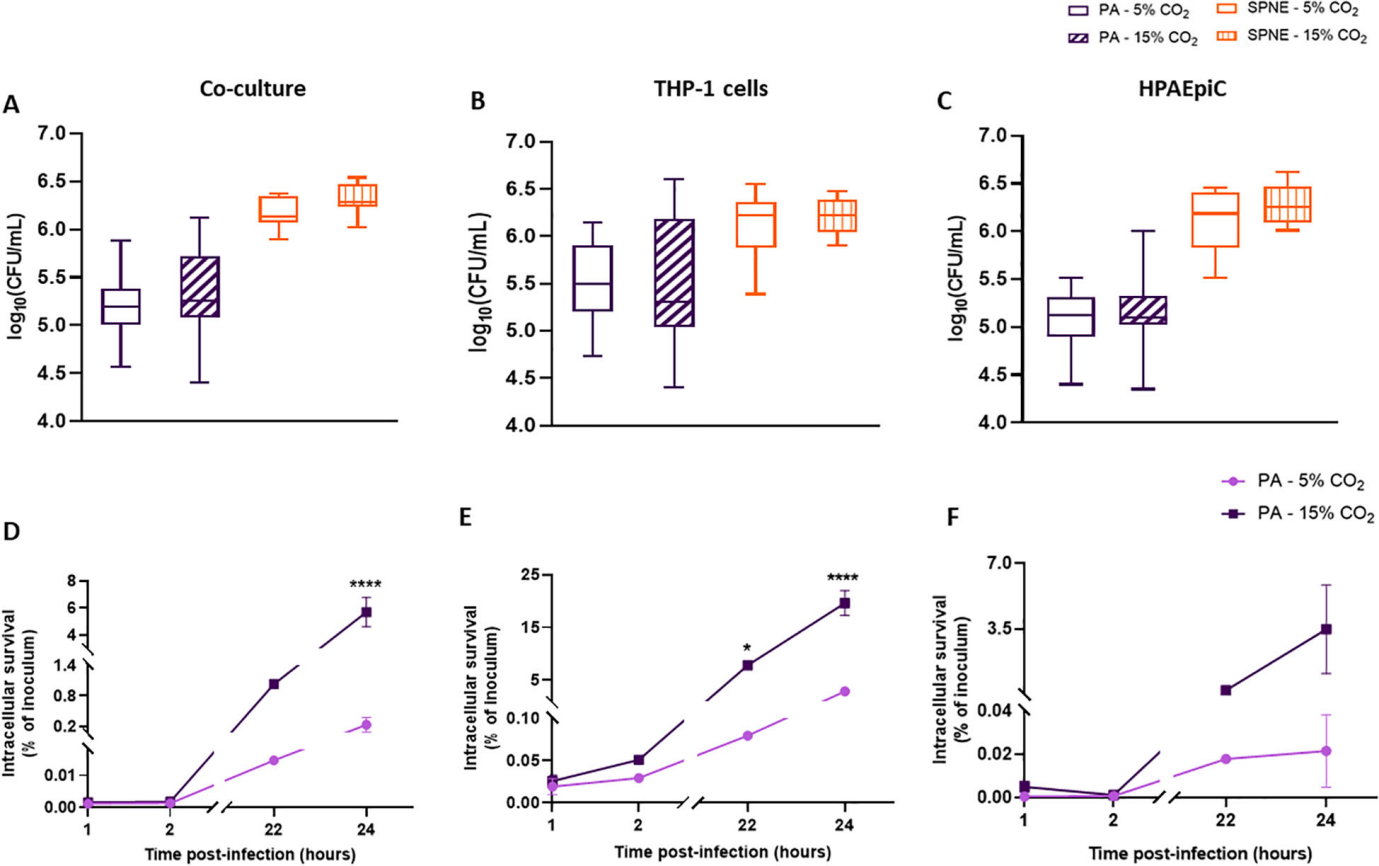
Extracellular and intracellular bacterial survival of *P. aeruginosa* in the infected co-culture, macrophage-like THP-1 cells and HPAEpiC under normocapnic and hypercapnic acidosis conditions. Extracellular bacterial survival of *P. aeruginosa* (PA) and *S. pneumoniae* (SPNE) immediately at the end of infection in the HPAEpiC and macrophage-like THP-1 cells co-culture, macrophage-like THP-1 cell culture, and HPAEpiC **(A, B, C)**. Intracellular bacterial survival of *P. aeruginosa* in the HPAEpiC and macrophage-like THP-1 cells co-culture, macrophage-like THP-1 cell culture, and HPAEpiC 1, 2, 22 and 24 h post-infection compared to the initial inoculum administered **(D, E, F)**. n=12-15 in extracellular studies and n= 3-5 in intracellular studies. Data represented as mean ± SEM. * p≤0.05; **** p≤0.0001.

One hour post-infection, no significant differences in extracellular bacterial load clearance were determined between normocapnic and HCA conditions in the co-culture, macrophage-like THP-1 cells and HPAEpiC cultures (**Figures 5A–C**).

Intracellular *P. aeruginosa* was detected in lysates from all culture types (**Figures 5D–F**), with the macrophage-like THP-1 cells harbouring the highest bacterial load (**Figure 5E**). HCA significantly increased the bacterial load in the co-culture and macrophage-like THP-1 cells. Specifically, in the co-culture model, the bacterial load of *P. aeruginosa* reached 5.69% of the initial inoculum under HCA and 0.23% under normocapnia (**Figure 5D**). In *P. aeruginosa*-infected macrophage-like THP-1 cultures, the intracellular bacterial load reached 19.61% of the initial inoculum under HCA and only 2.88% under normocapnia (**Figure 5E**). Although the increase was not statistically significant, a higher bacterial load was also found in HPAEpiC under HCA (**Figure 5F**).

*S. pneumoniae* CFUs were not detected in cell lysates at 2, 22, or 24 hours post-infection, even when the study was performed with p/s or vancomycin washes. However, at 1 hour post-infection, bacteria were detected in macrophage-like THP-1 cells, with no significant differences between CO_2_ conditions.

## 4 Discussion

In the present study, we demonstrate that HCA induces pathogen-specific changes that prolong exacerbated lung inflammation, mainly in macrophage-like cells. In addition, prolonged hypercapnia also compromises epithelial barrier integrity by decreasing the tight junction expression. These combined effects are likely to create a permissive environment for bacterial persistence.

We have developed a robust co-culture model of alveolar epithelial cells and macrophage-like cells to assess the effects of HCA on infection with *P. aeruginosa* and *S. pneumoniae*, two clinically relevant pathogens (Cillóniz et al., 2011; Torres et al., 2021; GBD 2021 Lower Respiratory Infections and Antimicrobial Resistance Collaborators, 2024; WHO bacterial priority pathogens list, 2024). This approach provides a valuable tool for studying early host-pathogen interactions and the potential role of hypercapnia in the alveolar microenvironment.

A key finding is that HCA has different effects on IL-1β secretion depending on the pathogen. In *S. pneumoniae*-infected cultures, IL-1β secretion is CO_2_-dependent, whereas *P. aeruginosa* strongly induces IL-1β production regardless of CO_2_, although HCA appears to promote its release. These findings are consistent with previous studies in which hypercapnia increased IL-1β production in the lung of animal models exposed to LPS or *E. coli* infection compared to controls (Nichol et al., 2009; Norozian et al., 2011).

Differences in IL-1β secretion under hypercapnia might result from its selective modulation of inflammatory pathways. To reach its mature form, IL-1β is processed by the inflammasome, and hypercapnia has been reported to activate the NLRP3 inflammasome (Ding et al., 2018; Casalino-Matsuda et al., 2021), inducing IL-1β overproduction (Ding et al., 2018). However, widely reported studies suggest that hypercapnia affects key components in the NF-κB signalling pathway, leading to its inhibition and, consequently, the regulation of proinflammatory pathways such as IL-1β transcription (Takeshita et al., 2003; Helenius et al., 2009; Cummins et al., 2010; Casalino-Matsuda et al., 2021). In line with this, previous studies conducted in macrophages have described a CO_2_ concentration-dependent decrease in pro-inflammatory markers (Lang et al., 2005; Wang et al., 2010). This apparent contradiction suggests that other non-canonical pathways converging on NF-κB activation, or even NF-κB-independent pathways such as HIF-1α, might be activated under hypercapnic conditions and lead to increased IL-1β production.

Our studies also indicate that hypercapnia increases the release of chemoattractant mediators at 1-hour post-infection but does not alter their production at later stages. In our model, macrophage-like THP-1 cells are the highest producers of IL-1β and chemoattractant mediators since the onset of the infection. Their strong inflammatory response suggests their potential role in shaping the inflammatory profile in co-culture conditions. At later stages of the infection, alveolar epithelial cells play a central role in inflammation and might influence the chemoattractant mediators released by macrophage-like THP-1 cells in the co-culture, as suggested by the IL-8 secretion profile at 24 hours post-infection, which more closely resembles that of HPAEpiC than macrophage-like THP-1 cells. However, the interaction of these cellular types might not be dependent on the CO_2_ exposure.

Hypercapnia also altered tight junction expression. At 1 hour post-infection, both pathogens induced an upregulation of ZO-1 and occludin under hypercapnic conditions—*S. pneumoniae* in both co-cultures and monocultures, and *P. aeruginosa* mainly in co-cultures—suggesting a potential acute response to infection, in which macrophages might be essential to upregulate the tight junctions of the epithelial cells particularly infected by *P. aeruginosa*. Cytokines are known to regulate the tight junctions through different signaling pathways (Capaldo and Nusrat, 2009), which might explain the early upregulation of ZO-1 and occludin as a part of a reactive response. By 24 hours, this pattern was not maintained: occludin expression significantly decreased under hypercapnia, while ZO-1 remained unchanged. This selective reduction, possibly driven by redox-sensitive mechanisms (Walter et al., 2009), might hinder epithelial barrier restoration and increase susceptibility to tissue damage and reinfection. These findings are consistent with previous research in a non-infected model where HCA impaired pulmonary tissue repair by reducing epithelial cell proliferation and migration via an NF-κB dependent mechanism (O’Toole et al., 2009).

Structural damage, together with hypercapnia-exacerbated inflammation, might create an environment that compromises host cell function and influences bacterial burden. In our study, hypercapnia selectively enhanced the intracellular replication of *P. aeruginosa* within macrophages-like THP-1 cells in a time-dependent manner.

The early presence of intracellular bacteria in macrophages suggests phagocytosis, but hypercapnia affected their intracellular burden in a pathogen-specific manner. *S. pneumoniae* was not affected by HCA and was only found intracellularly early after infection, suggesting macrophage phagocytosis. However, *P. aeruginosa* remained intracellular and its load increased over time, indicating active replication and bacterial persistance within macrophages under hypercapnia. These findings align with previous studies reporting that hypercapnia reduces the phagocytosis of macrophages primed with toll-like receptor agonists such as LPS (Wang et al., 2010) without significantly affecting extracellular bacterial growth (Helenius et al., 2009; Nichol et al., 2009). Autophagy is known to play a critical role in eliminating bacterial intracellular niches (Huang and Brumell, 2014), and hypercapnia has been described to increase bacterial survival and impair bacterial killing by reducing *P. aeruginosa*-induced autophagy in THP-1 macrophages (Casalino-Matsuda et al., 2015). This might explain our findings of enhanced *P. aeruginosa* replication under hypercapnic conditions.

Here we describe for the first time the intracellular replication of *P. aeruginosa* in alveolar epithelial cells under HCA. Although intracellular bacteria had previously been reported under normocapnia (Garcia-Medina et al., 2005), HCA did not alter the bacterial load, despite the deleterious and prolonged effects of hypercapnia on alveolar epithelial cell integrity. Also, no differences in extracellular survival of *P. aeruginosa* or *S. pneumoniae* were found between CO_2_ conditions.

The intracellular life of *P. aeruginosa* in lung infections might have been overlooked, as this pathogen has traditionally been considered to be extracellular. However, its presence in the lung epithelium of cystic fibrosis patients (Crabbé, 2024) highlights a potential role in chronic and recurrent acute infection. Intracellular persistence, together with biofilm formation and structural lung damage, might contribute to chronic diseases often associated with hypercapnia such as chronic obstructive pulmonary disease or bronchiectasis.

In spite of the strengths previously mentioned, our study presents limitations and future directions. The co-culture model does not fully capture *in vivo* immune complexity, and future studies could benefit from 3D models. Moreover, broadening the range of pathogens used in future studies could provide valuable insights into whether the observed effects are pathogen-specific or more generally applicable to respiratory infections. In addition, experimental designs incorporating moderate CO_2_ concentrations could provide the threshold at which hypercapnia begins to affect immune responses and its temporal dynamics. Additionally, it is important to note that our study specifically addressed HCA, and that attempts to buffer and stabilize the pH of culture medium resulted in persistent acidification. Although many studies have described that the effects of hypercapnia are pH-independent (Helenius et al., 2009; O’Toole et al., 2009; Cummins et al., 2010; Wang et al., 2010; Casalino-Matsuda et al., 2015), our experimental approach could not fully dissociate these parameters. Finally, statistical differences between LME and t-test outcomes highlight the importance of using multiple methods for data interpretation.

Clinically, our findings might have important implications for the management and prognostic of patients with respiratory infections under elevated CO_2_, including those receiving mechanical ventilation or with chronic lung disease. Unraveling this immune modulation under an infectious context could lead to new prognostic or therapeutic strategies. These range from optimizing ventilation and extracorporeal CO_2_ removal (Maamar et al., 2023), to targeting specific molecular mechanism, such as the use of DNMT3a inhibitors to reverse the CO_2_-driven hypermethylation of the *CDH1* gene (Yeung-Luk et al., 2024). Such approaches combined with antibiotics (Osorio-Rodríguez et al., 2025) might be clinically relevant to mitigate the detrimental effects of high CO_2_ on infection outcomes, such as bacterial persistence and delayed barrier repair.

## Conclusions

The present study highlights the importance of HCA effects in a model of respiratory cellular infection. Here, we demonstrate that HCA condition differentially influence host responses to *P. aeruginosa* and *S. pneumoniae* in a pathogen-specific manner, compromising the barrier function and enhancing lung inflammation, particularly over prolonged exposure. Macrophages could play a dual role by contributing to early inflammation and tight junction regulation, while also serving as a niche for intracellular bacterial persistence under HCA conditions. These findings emphasize the relevance of CO_2_-driven immune dysregulation in lung infection pathophysiology and highlight the value of this model for exploring novel therapeutic strategies.

## Data Availability

The raw data supporting the conclusions of this article will be made available by the authors, without undue reservation.
