# Supplementary material for: Hypercapnic acidosis affects in vitro lung infection response in a pathogen-specific manner
Source: Front Cell Infect Microbiol. 2026 Feb 3;15:1708427. doi: 10.3389/fcimb.2025.1708427 (PMC12909561; doi:10.3389/fcimb.2025.1708427)
Supplement: Supplementary file 1 [file SupplementaryFile1.docx]

**Supplementary Material**

**S1. Detailed cell culture conditions**

THP-1 cells (American Type Culture Collection, Virginia, USA), a human monocytic cell line, and human pulmonary alveolar epithelial cells (HPAEpiC) (Innoprot, Bizkaia, Spain) were seeded in 24-well plates, either in monoculture or co-culture (Figure 1). In monocultures, THP-1 cells and HPAEpiC were seeded at a final concentration of 200,000 and 100,000 cells per well, respectively. The co-culture was established at a 1:5 ratio of THP-1 to HPAEpiC (Fritz et al., 2011), resulting in a final concentration of 100,000 cells per well. Cell viability was determined by trypan blue staining.

THP-1 monocytic cells (American Type Culture Collection, Virginia, USA) were cultured in RPMI 1640 medium (Biowest, Nuaillé, France) supplemented with 10% heat-inactivated fetal bovine serum (FBS) (Innoprot, Bizkaia, Spain), 1% penicillin-streptomycin (p/s) (Capricorn Scientific, Ebsdorfergrund, Germany), 1% amphotericin (Biowest), and 0.5% L-glutamine (Capricorn Scientific). To induce differentiation into macrophage-like cells, THP-1 were treated with 100 ng/mL of PMA (Sigma-Aldrich, St. Louis, MO, USA) for 48 hours. After differentiation, the cells were washed and maintained in AEpiCM (Innoprot, Bizkaia, Spain) supplemented with 2% FBS, 1% Epithelial Cell Growth Supplement (EpiCGS) (Innoprot, Bizkaia, Spain), and 1% p/s for an additional 24 hours to achieve the non-activated macrophage phenotype (M0). Five hours before infection, the macrophage-like THP-1 cells were primed with 2 µg/mL LPS from *Escherichia coli* O55:B5 (Sigma-Aldrich) diluted in AEpiCM to polarize them towards a proinflammatory M1 phenotype.

HPAEpiC used in the experiments corresponded to passages 4 to 6. Cultures were grown on poly-L-lysine-coated flasks, and when 90% confluence was reached, trypsinization was performed and cultures were used for the experiment. HPAEpiC were cultured in AEpiCM supplemented with 2% inactivated FBS, 1% EpiCGS and 1% p/s in 24-well and plates precoated with poly-L-lysine solution (Sigma-Aldrich, St. Louis, MO, USA). Cells were seeded either individually or in co-culture with macrophage-like THP-1 cells seeded 48 hours earlier. Cultures were left to rest for 24 hours.

**S2. Bacterial culture and infection protocol**

*Pseudomonas aeruginosa* (strain PAO1) was grown in Luria Broth (LB) medium (Gibco, Thermo Fisher Scientific, Waltham, MA, USA) to log phase (≈109 CFU P. aeruginosa/ml, OD600 0.45-0.55). *Streptococcus pneumoniae* (serotype 19A) (Amaro et al., 2021) was grown in THY medium (Todd Hewitt broth medium with 2% yeast extract) (Sigma-Aldrich, St. Louis, MO, USA) to log phase (≈107 CFU *S. pneumoniae*/ml, OD600 0.45-0.60). Bacteria were centrifuged and resuspended in fresh AEpiCM with a MOI of 1:50 for *P. aeruginosa*-infected cultures and 1:20 for *S. pneumoniae*-infected cultures. MOI values were previously determined by performing growth curves of each bacterial strain and selected for eliciting a robust inflammatory response without causing excessive cell death (data not shown). Both pathogens have proved lung infection capacity in experimental models previously described (19,20). Cultures containing medium without bacteria were also included as controls. Infections were performed for 1 hour at 37°C under normocapnic (5% CO2, pH 7,3) or HCA (15% CO2, pH 7) conditions. After the 1-hour infection, cultures were washed three times with PBS containing 5X p/s, and then fresh AEpiCM supplemented with 1% EpiCGS and 1% p/s was added. Samples were collected at 1 or 24 hours post-infection to obtain cell supernatant and total intracellular protein.

**S3. Preparation of cell supernatants and intracellular extracts**

Cell culture supernatants were collected at 1 or 24 hours post-infection and centrifuged at 500 *x g* for 5 minutes at RT to remove debris. The protein concentrations of proinflammatory IL-1β (DY201-05), and chemoattractant CCL2 (DY279-05) and IL-8 (DY208-05) mediators were measured using ELISA kits according to the supplier’s protocol (R&D Systems, Minneapolis, USA).

Once the supernatant was removed, a solution containing protease inhibitor cocktail (Roche, Basel, Switzerland), sodium orthovanadate, and lysis buffer (10% Triton-X100, 3M NaCl, 300 mM Tris, 20 mM CaCl, 1M MgCl2) was added and the cells were scraped. The homogenate was incubated for 30 minutes on ice and then centrifuged at 16,500 x g for 20 minutes at 4ºC. The supernatant was collected and stored at -80ºC for subsequent analysis. Total intracellular protein was quantified using the BCA Pierce assay kit (Thermo Fisher Scientific, Waltham, MA, USA), and intracellular protein levels for the zonula occludens 1 (ZO-1) (EH15434) and occludin (EH1674) were analysed by ELISA according to the supplier’s protocol (FineTest, Boulder, CO, USA) in HPAEpiC cultures, both alone and in co-culture with macrophage-likeTHP-1 cells.

**S4. Analysis of bacterial survival**

After the 1-hour infection, the cell culture supernatant was seeded onto blood agar plates for subsequent analysis of the remaining extracellular bacteria post-infection. To determine the intracellular bacterial load, the cultures underwent three washes with PBS containing 5X p/s, gentamicin (25 μg/ml), or vancomycin (50 μg/ml). Cells were then lysed by adding cold distilled water for 10 minutes at different time points (1, 2, 22, and 24 hours post-infection). The resulting supernatant was seeded on blood agar plates and colony forming units (CFU) were counted.

**S5. Statistical analysis**

The Shapiro-Wilk test was used to evaluate the normality of the data. Depending on the distribution, either a t-test or a Mann-Whitney test was applied to compare the experimental groups. The following pairwise comparisons were conducted: Control 5% CO_2_ vs. Control 15% CO_2_, Control 5% CO_2_ vs. *P. aeruginosa*-infected 5% CO_2_, Control 5% CO_2_ vs. *S. pneumoniae*-infected 5% CO_2,_ Control 15% CO_2_ vs. *P. aeruginosa*-infected 15% CO_2,_ Control 15% CO_2_ vs. *S. pneumoniae*-infected 15% CO_2,_ *P. aeruginosa*-infected 5% CO_2_ vs. *P. aeruginosa*-infected 15% CO_2,_ and *S. pneumoniae*-infected 5% CO_2_ vs. *S. pneumoniae*-infected 15% CO_2_. Results were expressed as the geometric mean ± standard error of the mean (SEM). Linear mixed effects (LME) models were used to assess the fixed effect of different CO_2_ levels and bacterial infection, while variability between replicates and experiments was evaluated as a random effect. All analyses were performed using GraphPad Prism 8 and R (v.4.3.0), with RStudio (2024.12.1 Build 563). A *p*-value ≤0.05 was considered statistically significant.

**Additional Table 1. Effects of CO_2_ conditions and bacterial infection on cytokine levels expressed by co-culture and macrophage-like THP-1 cells 1 hour after infection.**

| **1h co-culture, IL-1β - Summary of Linear Mixed Effects Models** | | | | | | | | |
| --- | --- | --- | --- | --- | --- | --- | --- | --- |
| **Model** | effect | term | Beta-estimate | std.error | statistic | p.value | Conf Interval 2.5% | Conf Interval 97.5% |
| **Model** | Fixed | (Intercept) | 46.226 | 16.954 | 2.727 | 0.0126 | 12.996 | 79.456 |
| **Model** | Fixed | CO2 | -1.205 | 1.332 | -0.904 | 0.3760 | -3.816 | 1.406 |
| **Model** | Fixed | CO2:InfecciónPA | 3.019 | 1.883 | 1.603 | 0.1238 | -0.672 | 6.710 |
| **Model** | Fixed | CO2:InfecciónSP | 2.240 | 1.908 | 1.174 | 0.2536 | -1.500 | 5.980 |
| **Model** | Fixed | InfecciónPA | -16.978 | 22.264 | -0.763 | 0.4542 | -60.615 | 26.659 |
| **Model** | Fixed | InfecciónSP | -13.538 | 23.557 | -0.575 | 0.5716 | -59.710 | 32.634 |
| **Model** | Random | (Intercept) | 7.537 |  |  |  |  |  |
| **Model** | Random | Residual | 21.053 |  |  |  |  |  |

| **1h co-culture, IL-8 - Summary of Linear Mixed Effects Models** | | | | | | | | |
| --- | --- | --- | --- | --- | --- | --- | --- | --- |
| **Model** | effect | term | Beta-estimate | std.error | statistic | p.value | Conf Interval 2.5% | Conf Interval 97.5% |
| **Model** | Fixed | (Intercept) | 336.905 | 109.648 | 3.073 | 0.00493 | 121.995 | 551.815 |
| **Model** | Fixed | CO2 | -3.454 | 8.624 | -0.400 | 0.69207 | -20.357 | 13.449 |
| **Model** | Fixed | CO2:InfecciónPA | 25.048 | 14.225 | 1.761 | 0.09003 | -2.833 | 52.929 |
| **Model** | Fixed | CO2:InfecciónSP | 22.968 | 12.888 | 1.782 | 0.08641 | -2.292 | 48.228 |
| **Model** | Fixed | InfecciónPA | -325.434 | 156.222 | -2.083 | 0.04721 | -631.629 | -19.239 |
| **Model** | Fixed | InfecciónSP | -115.582 | 149.469 | -0.773 | 0.44633 | -408.541 | 177.377 |
| **Model** | Random | (Intercept) | 98.820 |  |  |  |  |  |
| **Model** | Random | Residual | 165.880 |  |  |  |  |  |

| **1h co-culture, CCL2 - Summary of Linear Mixed Effects Models** | | | | | | | | |
| --- | --- | --- | --- | --- | --- | --- | --- | --- |
| **Model** | effect | term | Beta- estimate | std.error | statistic | p.value | Conf Interval 2.5% | Conf Interval 97.5% |
| **Model** | Fixed | (Intercept) | 150.545 | 52.156 | 2.886 | 0.00913 | 48.319 | 252.771 |
| **Model** | Fixed | CO2 | 0.307 | 4.665 | 0.066 | 0.94821 | -8.836 | 9.450 |
| **Model** | Fixed | CO2:InfecciónPA | 0.470 | 7.151 | 0.066 | 0.94823 | -13.546 | 14.486 |
| **Model** | Fixed | CO2:InfecciónSP | 5.555 | 7.376 | 0.753 | 0.46016 | -8.902 | 20.012 |
| **Model** | Fixed | InfecciónPA | -35.086 | 77.882 | -0.451 | 0.65719 | -187.735 | 117.563 |
| **Model** | Fixed | InfecciónSP | -39.955 | 82.463 | -0.485 | 0.63329 | -201.582 | 121.672 |
| **Model** | Random | (Intercept) | 0.866 |  |  |  |  |  |
| **Model** | Random | Residual | 80.796 |  |  |  |  |  |

| **1h macrophage-like THP-1, IL-1β - Summary of Linear Mixed Effects Models** | | | | | | | | |
| --- | --- | --- | --- | --- | --- | --- | --- | --- |
| **Model** | effect | term | Beta-estimate | std.error | statistic | p.value | Conf Interval 2.5% | Conf Interval 97.5% |
| **Model** | Fixed | (Intercept) | 35.752 | 53.893 | 0.663 | 0.51652 | -69.878 | 141.382 |
| **Model** | Fixed | CO2 | 1.263 | 4.260 | 0.296 | 0.77067 | -7.087 | 9.613 |
| **Model** | Fixed | CO2:InfecciónPA | -0.345 | 6.156 | -0.056 | 0.95604 | -12.411 | 11.721 |
| **Model** | Fixed | CO2:InfecciónSP | 16.548 | 6.156 | 2.688 | 0.01617 | 4.482 | 28.614 |
| **Model** | Fixed | InfecciónPA | 228.986 | 70.394 | 3.253 | 0.00499 | 91.014 | 366.958 |
| **Model** | Fixed | InfecciónSP | -21.736 | 71.349 | -0.305 | 0.76457 | -161.580 | 118.108 |
| **Model** | Random | (Intercept) | 37.721 |  |  |  |  |  |
| **Model** | Random | Residual | 62.856 |  |  |  |  |  |

| **1h macrophage-like THP-1, IL-8 - Summary of Linear Mixed Effects Models** | | | | | | | | |
| --- | --- | --- | --- | --- | --- | --- | --- | --- |
| **Model** | effect | term | Beta-estimate | std.error | statistic | p.value | Conf Interval 2.5% | Conf Interval 97.5% |
| **Model** | Fixed | (Intercept) | 835.443 | 549.298 | 1.521 | 0.1447 | -241.181 | 1,912.067 |
| **Model** | Fixed | CO2 | 91.858 | 49.054 | 1.873 | 0.0766 | -4.288 | 188.004 |
| **Model** | Fixed | CO2:InfecciónPA | -94.229 | 73.580 | -1.281 | 0.2157 | -238.446 | 49.988 |
| **Model** | Fixed | CO2:InfecciónSP | -101.273 | 69.372 | -1.460 | 0.1607 | -237.242 | 34.696 |
| **Model** | Fixed | InfecciónPA | 1,582.240 | 822.965 | 1.923 | 0.0697 | -30.771 | 3,195.251 |
| **Model** | Fixed | InfecciónSP | 2,077.616 | 775.945 | 2.678 | 0.0149 | 556.764 | 3,598.468 |
| **Model** | Random | (Intercept) | 58.225 |  |  |  |  |  |
| **Model** | Random | Residual | 775.606 |  |  |  |  |  |

| **1h macrophage-like THP-1, CCL2 - Summary of Linear Mixed Effects Models** | | | | | | | | |
| --- | --- | --- | --- | --- | --- | --- | --- | --- |
| **Model** | effect | term | Beta-estimate | std.error | statistic | p.value | Conf Interval 2.5% | Conf Interval 97.5% |
| **Model** | Fixed | (Intercept) | 188.225 | 110.756 | 1.699 | 0.106 | -28.857 | 405.307 |
| **Model** | Fixed | CO2 | -1.332 | 8.063 | -0.165 | 0.871 | -17.135 | 14.471 |
| **Model** | Fixed | CO2:InfecciónPA | -4.473 | 11.645 | -0.384 | 0.705 | -27.297 | 18.351 |
| **Model** | Fixed | CO2:InfecciónSP | 5.942 | 11.022 | 0.539 | 0.596 | -15.661 | 27.545 |
| **Model** | Fixed | InfecciónPA | 84.022 | 133.226 | 0.631 | 0.536 | -177.101 | 345.145 |
| **Model** | Fixed | InfecciónSP | 49.068 | 130.355 | 0.376 | 0.711 | -206.428 | 304.564 |
| **Model** | Random | (Intercept) | 111.440 |  |  |  |  |  |
| **Model** | Random | Residual | 118.825 |  |  |  |  |  |

Beta coefficients are estimated from linear mixed-effects (LME) models including CO₂ exposure, *P. aeruginosa* infection, *S. pneumoniae* infection, and their interaction terms as fixed effects. CO₂ was entered as a numeric covariate, whereas *P. aeruginosa* and *S. pneumoniae* infections were included as factor variables. The models also included random effects to account for variability among biological replicates. Values are reported as beta estimates with 95% confidence intervals. n = 4-6 for each condition. Significance codes: p≤0.1; *p ≤ 0.05; **p ≤ 0.01; ***p ≤ 0.001; ****p ≤ 0.0001.

**Additional Table 2. Effects of CO_2_ conditions and bacterial infection on cytokine levels expressed by co-culture, macrophage-like THP-1 cells and HPAEpiC 24 hours after infection.**

| **24h co-culture, IL-1β - Summary of Linear Mixed Effects Models** | | | | | | | | |
| --- | --- | --- | --- | --- | --- | --- | --- | --- |
| **model** | effect | term | Beta-estimate | std.error | statistic | p.value | Conf Interval 2.5% | Conf Interval 97.5% |
| **model** | Fixed | (Intercept) | 8.894 | 33.920 | 0.262 | 0.793987 | -57.589 | 75.377 |
| **model** | Fixed | CO2 | 0.643 | 2.726 | 0.236 | 0.814266 | -4.700 | 5.986 |
| **model** | Fixed | CO2:InfecciónPA | 6.957 | 5.012 | 1.388 | 0.169809 | -2.867 | 16.781 |
| **model** | Fixed | CO2:InfecciónSP | 0.865 | 4.832 | 0.179 | 0.858453 | -8.606 | 10.336 |
| **model** | Fixed | InfecciónPA | 227.247 | 55.954 | 4.061 | 0.000132 | 117.577 | 336.917 |
| **model** | Fixed | InfecciónSP | -10.167 | 54.082 | -0.188 | 0.851465 | -116.168 | 95.834 |
| **model** | Random | (Intercept) | 48.739 |  |  |  |  |  |
| **model** | Random | Residual | 89.225 |  |  |  |  |  |

| **24h co-culture, IL-8 - Summary of Linear Mixed Effects Models** | | | | | | | | |
| --- | --- | --- | --- | --- | --- | --- | --- | --- |
| **model** | effect | term | Beta-estimate | std.error | statistic | p.value | Conf Interval 2.5% | Conf Interval 97.5% |
| **model** | Fixed | (Intercept) | 2,436.610 | 653.595 | 3.728 | 0.000392 | 1,155.564 | 3,717.656 |
| **model** | Fixed | CO2 | -17.962 | 41.917 | -0.429 | 0.669618 | -100.119 | 64.195 |
| **model** | Fixed | CO2:InfecciónPA | -4.678 | 77.794 | -0.060 | 0.952222 | -157.154 | 147.798 |
| **model** | Fixed | CO2:InfecciónSP | -11.253 | 72.602 | -0.155 | 0.877275 | -153.553 | 131.047 |
| **model** | Fixed | InfecciónPA | 731.297 | 871.686 | 0.839 | 0.404397 | -977.208 | 2,439.802 |
| **model** | Fixed | InfecciónSP | 591.500 | 813.637 | 0.727 | 0.469696 | -1,003.229 | 2,186.229 |
| **model** | Random | (Intercept) | 1,436.595 |  |  |  |  |  |
| **model** | Random | Residual | 1,390.225 |  |  |  |  |  |

| **24h co-culture, CCL2 - Summary of Linear Mixed Effects Models** | | | | | | | | |
| --- | --- | --- | --- | --- | --- | --- | --- | --- |
| **model** | effect | term | Beta-estimate | std.error | statistic | p.value | Conf Interval 2.5% | Conf Interval 97.5% |
| **model** | Fixed | (Intercept) | 77.242 | 16.514 | 4.677 | 1.4e-05 | 44.875 | 109.609 |
| **model** | Fixed | CO2 | -2.003 | 0.973 | -2.059 | 0.0433 | -3.910 | -0.096 |
| **model** | Fixed | CO2:InfecciónPA | 1.012 | 1.805 | 0.560 | 0.5770 | -2.526 | 4.550 |
| **model** | Fixed | CO2:InfecciónSP | 0.973 | 1.685 | 0.578 | 0.5654 | -2.330 | 4.276 |
| **model** | Fixed | InfecciónPA | -5.632 | 20.226 | -0.278 | 0.7815 | -45.275 | 34.011 |
| **model** | Fixed | InfecciónSP | 0.063 | 18.879 | 0.003 | 0.9974 | -36.940 | 37.066 |
| **model** | Random | (Intercept) | 39.222 |  |  |  |  |  |
| **model** | Random | Residual | 32.256 |  |  |  |  |  |

| **24h macrophage-like THP-1, IL-1β - Summary of Linear Mixed Effects Models** | | | | | | | | |
| --- | --- | --- | --- | --- | --- | --- | --- | --- |
| **Model** | effect | term | Beta-estimate | std.error | statistic | p.value | Conf Interval 2.5% | Conf Interval 97.5% |
| **Model** | Fixed | (Intercept) | 163.838 | 65.451 | 2.503 | 0.0145 | 35.554 | 292.122 |
| **Model** | Fixed | CO2 | 15.973 | 3.717 | 4.297 | 5.26e-05 | 8.688 | 23.258 |
| **Model** | Fixed | CO2:InfecciónPA | -15.419 | 6.752 | -2.284 | 0.0253 | -28.653 | -2.185 |
| **Model** | Fixed | CO2:InfecciónSP | -0.498 | 6.348 | -0.078 | 0.9377 | -12.940 | 11.944 |
| **Model** | Fixed | InfecciónPA | 505.211 | 75.822 | 6.663 | 4.36e-09 | 356.600 | 653.822 |
| **Model** | Fixed | InfecciónSP | -4.145 | 71.111 | -0.058 | 0.9537 | -143.523 | 135.233 |
| **Model** | Random | (Intercept) | 174.959 |  |  |  |  |  |
| **Model** | Random | Residual | 126.046 |  |  |  |  |  |

| **24h macrophage-like THP-1, IL-8 - Summary of Linear Mixed Effects Models** | | | | | | | | |
| --- | --- | --- | --- | --- | --- | --- | --- | --- |
| **Model** | effect | term | Beta-estimate | std.error | statistic | p.value | Conf Interval 2.5% | Conf Interval 97.5% |
| **Model** | Fixed | (Intercept) | 5,386.173 | 1,129.930 | 4.767 | 9.3e-06 | 3,171.510 | 7,600.836 |
| **Model** | Fixed | CO2 | -65.114 | 72.762 | -0.895 | 0.374 | -207.728 | 77.500 |
| **Model** | Fixed | CO2:InfecciónPA | 43.192 | 132.179 | 0.327 | 0.745 | -215.879 | 302.263 |
| **Model** | Fixed | CO2:InfecciónSP | -71.877 | 124.265 | -0.578 | 0.565 | -315.436 | 171.682 |
| **Model** | Fixed | InfecciónPA | -2,148.882 | 1,483.984 | -1.448 | 0.152 | -5,057.491 | 759.727 |
| **Model** | Fixed | InfecciónSP | 1,602.747 | 1,391.947 | 1.151 | 0.253 | -1,125.469 | 4,330.963 |
| **Model** | Random | (Intercept) | 2,711.712 |  |  |  |  |  |
| **Model** | Random | Residual | 2,467.486 |  |  |  |  |  |

| **24h macrophage-like THP-1, CCL2- Summary of Linear Mixed Effects Models** | | | | | | | | |
| --- | --- | --- | --- | --- | --- | --- | --- | --- |
| **Model** | effect | term | Beta-estimate | std.error | statistic | p.value | Conf Interval 2.5% | Conf Interval 97.5% |
| **Model** | Fixed | (Intercept) | 402.920 | 109.943 | 3.665 | 0.000466 | 187.432 | 618.408 |
| **Model** | Fixed | CO2 | -4.404 | 9.057 | -0.486 | 0.628250 | -22.156 | 13.348 |
| **Model** | Fixed | CO2:InfecciónPA | -14.352 | 16.453 | -0.872 | 0.385923 | -46.600 | 17.896 |
| **Model** | Fixed | CO2:InfecciónSP | -13.436 | 15.468 | -0.869 | 0.387902 | -43.753 | 16.881 |
| **Model** | Fixed | InfecciónPA | 55.350 | 184.450 | 0.300 | 0.764968 | -306.172 | 416.872 |
| **Model** | Fixed | InfecciónSP | 145.132 | 173.153 | 0.838 | 0.404672 | -194.248 | 484.512 |
| **Model** | Random | (Intercept) | 147.527 |  |  |  |  |  |
| **Model** | Random | Residual | 307.146 |  |  |  |  |  |

| **24h HPAEpiC, IL-8 - Summary of Linear Mixed Effects Models** | | | | | | | | |
| --- | --- | --- | --- | --- | --- | --- | --- | --- |
| **Model** | effect | term | Beta-estimate | std.error | statistic | p.value | Conf Interval 2.5% | Conf Interval 97.5% |
| **Model** | Fixed | (Intercept) | 219.777 | 118.332 | 1.857 | 0.0675 | -12.154 | 451.708 |
| **Model** | Fixed | CO2 | -12.385 | 10.580 | -1.171 | 0.2458 | -33.122 | 8.352 |
| **Model** | Fixed | CO2:InfecciónPA | 4.070 | 19.636 | 0.207 | 0.8364 | -34.417 | 42.557 |
| **Model** | Fixed | CO2:InfecciónSP | 12.724 | 18.325 | 0.694 | 0.4898 | -23.193 | 48.641 |
| **Model** | Fixed | InfecciónPA | 205.364 | 219.538 | 0.935 | 0.3528 | -224.930 | 635.658 |
| **Model** | Fixed | InfecciónSP | -200.726 | 204.886 | -0.980 | 0.3307 | -602.303 | 200.851 |
| **Model** | Random | (Intercept) | 9.805 |  |  |  |  |  |
| **Model** | Random | Residual | 350.901 |  |  |  |  |  |

| **24h HPAEpiC, CCL2 - Summary of Linear Mixed Effects Models** | | | | | | | | |
| --- | --- | --- | --- | --- | --- | --- | --- | --- |
| **Model** | effect | term | Beta-estimate | std.error | statistic | p.value | Conf Interval 2.5% | Conf Interval 97.5% |
| **Model** | Fixed | (Intercept) | 6.154 | 5.896 | 1.044 | 0.30021 | -5.402 | 17.710 |
| **Model** | Fixed | CO2 | 0.063 | 0.504 | 0.125 | 0.90076 | -0.925 | 1.051 |
| **Model** | Fixed | CO2:InfecciónPA | -1.390 | 0.936 | -1.485 | 0.14214 | -3.225 | 0.445 |
| **Model** | Fixed | CO2:InfecciónSP | -0.488 | 0.874 | -0.558 | 0.57861 | -2.201 | 1.225 |
| **Model** | Fixed | InfecciónPA | 33.585 | 10.479 | 3.205 | 0.00205 | 13.046 | 54.124 |
| **Model** | Fixed | InfecciónSP | 5.782 | 9.781 | 0.591 | 0.55633 | -13.389 | 24.953 |
| **Model** | Random | (Intercept) | 5.341 |  |  |  |  |  |
| **Model** | Random | Residual | 16.731 |  |  |  |  |  |

Beta coefficients are estimated from linear mixed-effects (LME) models including CO₂ exposure, *P. aeruginosa* infection, *S. pneumoniae* infection, and their interaction terms as fixed effects. CO₂ was entered as a numeric covariate, whereas *P. aeruginosa* and *S. pneumoniae* infections were included as factor variables. The models also included random effects to account for variability among biological replicates. Values are reported as beta estimates with 95% confidence intervals. n = 9-12 for each condition. Significance codes: p≤0.1; *p ≤ 0.05; **p ≤ 0.01; ***p ≤ 0.001; ****p ≤ 0.0001.
